# Metabolic load during strength training or NMES in individuals with COPD: results from the *DICES* trial

**DOI:** 10.1186/1471-2466-14-146

**Published:** 2014-09-02

**Authors:** Maurice JH Sillen, Frits ME Franssen, Anouk W Vaes, Jeannet ML Delbressine, Emiel FM Wouters, Martijn A Spruit

**Affiliations:** 1 Department of Research & Education, CIRO+, centre of expertise for chronic organ failure, Hornerheide 1, Horn, the Netherlands; 2 Department of Respiratory Medicine, Maastricht University Medical Centre (MUMC+), Maastricht, the Netherlands

**Keywords:** Chronic obstructive pulmonary disease, Neuromuscular electrical stimulation, Pulmonary rehabilitation, Strength training

## Abstract

**Background:**

Strength training and neuromuscular electrical stimulation (NMES) are effective training modalities for improving muscle function, exercise performance and health status in individuals with COPD. The aim of the present study was to analyze the metabolic load of these training modalities at baseline, half-way, and at the end of an eight-week interdisciplinary pulmonary rehabilitation program in a subgroup of individuals with COPD of the ***DICES*** trial.

**Methods:**

Of 24 individuals with COPD (FEV_1_: 34 ± 2% predicted, men: 58%, age: 66 (61–68) years), peak oxygen uptake (VO_2_), peak minute ventilation (V_E_), heart rate, oxygen saturation and symptom scores were assessed during HF-NMES (75 Hz), LF-NMES (15 Hz) and strength training at three moments during their pulmonary rehabilitation program.

**Results:**

Intervention-related peak VO_2_ did not change over time during HF-NMES, LF-NMES or strength training. Intervention-related peak V_E_ did not change over time during strength training or LF-NMES and increased slightly, but significantly over time during HF-NMES. Peak VO_2_ and V_E_ were significantly higher during strength training compared to HF-NMES or LF-NMES. Oxygen saturation significantly decreased after the first measurements during HF-NMES and strength training group to baseline, while no significant changes in oxygen saturation were observed during the other measurements. Heart rate significantly increased compared to baseline in all groups at all moments and was significantly higher after strength training compared to HF-NMES or LF-NMES. Median end scores (points) for dyspnea, fatigue and muscle pain ranged from 1 to 3, from 0.5 to 2 and from 0 to 6 after HF-NMES, from 2 to 3, from 2 to 5 and from 0 to 9 after LF-NMES and from 2 to 5, from 1.5 to 4 and from 0 to 28 after strength training respectively.

**Conclusions:**

To conclude, the metabolic load and symptom scores remain acceptable low over time with increasing training loads during HF-NMES, LF-NMES or strength training.

**Trial registration:**

**Trial registration:**NTR2322

## Background

Individuals with chronic obstructive pulmonary disease (COPD) may suffer from lower-limb muscle weakness and poor exercise capacity, in particular those with severe to very severe dyspnea [1-3]. This is most probably due to reductions in weight-bearing daily physical activities [4]. Therefore, an exercise-based pulmonary rehabilitation program may be beneficial [5]. Severely dyspneic individuals with COPD (i.e., modified MRC dyspnea grade 3 or 4), however, are less likely to complete a pulmonary rehabilitation program [6]. This may be due to exercise-induced dyspnea, particularly during whole-body endurance training [7]. Therefore, strength training [8] or transcutaneous neuromuscular electrical stimulation (NMES) [9,10] may be preferential alternative rehabilitative modalities for severely dyspneic individuals with COPD [11,12]. These interventions are safe and effective in severely dyspneic individuals with COPD and quadriceps muscle weakness at baseline [13]. Indeed, lower-limb muscle function, functional exercise performance, problematic activities of daily life, mood status, and health status improved significantly following eight weeks of strength training, high-frequency (HF, 75 Hertz) NMES, or low-frequency (LF, 15 Hertz) NMES [13].

A major advantage of strength training and NMES is the relatively low metabolic load (e.g., the intervention-related peak oxygen uptake (VO_2_) and ventilation (V_E_)), accompanied with relatively low dyspnea symptom scores [14,15]. The metabolic load during multiple successive sessions of strength training has been reported once in 11 individuals with COPD [7]. The leg press strengthening exercise increased significantly during a 12-week pulmonary rehabilitation program (+43% of baseline training load), accompanied by a significant increase in metabolic load over time (+23% of baseline intervention-related peak VO_2_; +18% of baseline intervention-related peak V_E_) [7]. The metabolic load during a session of high-frequency (HF) or low-frequency (LF) NMES has only been measured cross-sectionally [14,15]. Whether and to what extent the metabolic load will remain stable over time while the NMES pulse amplitude is expected to increase [16] remains currently unknown in individuals with COPD. Moreover, the actual course of NMES pulse amplitude has never been described in individuals with COPD. This, however, will provide a better insight in the feasibility and efficacy of these types of local muscle training.

The aim of the present study was to analyze the metabolic load of the different local muscle training modalities at baseline, half-way, and at the end of the eight-week program in a subgroup of individuals with COPD who participated in the ***DICES*** (***D***yspneic ***I***ndividuals with ***C***OPD: ***E***lectrical stimulation or ***S***trength training) trial. *A priori*, we hypothesized that individuals with COPD are able to increase the strength training load or NMES pulse amplitude (irrespective of stimulation frequency), while the metabolic load will remain stable compared to baseline.

## Methods

### Participants

In the ***DICES*** trial, individuals with COPD with mMRC dyspnea grade 3 or 4 [2], and quadriceps weakness [17] were randomly assigned to lower-limb HF-NMES (75 Hertz), lower-limb LF-NMES (15 Hertz), or lower-limb strength training (leg extension strengthening exercise, and leg press strengthening exercise [8,18]). These interventions took place in group sessions, twice per day, 5 times per week for 8 weeks. All sessions were supervised by a physiotherapist. The interdisciplinary treatment was identical for all participants, and treadmill walking or stationary ergometry cycling was not applied during the trial. Symptom scores for dyspnea, fatigue, and muscle pain were assessed before and after each session [19].

The Medical Ethical Committee of the Maastricht University Medical Centre + (MEC 09-3-072) approved this trial, which conformed to the principles outlined in the World Medical Association declaration of Helsinki which was revised in Seoul [20]. Details of the trial were registered at http://www.trialregister.nl NTR2322) before first subject enrolment. All patients gave written informed consent to participate in the study and a subgroup additionally gave written informed consent to undergo the measurements of the metabolic load. Some of the baseline findings, and the efficacy data of the ***DICES*** trial have been published before [21,22].

#### Interventions

Please, *see* Additional file 1 for all details.

#### Outcomes

Outcome assessors were blinded for treatment allocation. The investigators supervising the interventions (MJHS, AWV) were blinded for the initial results, and were not involved in the outcome assessment. Participants were instructed to not divulge their group allocation. Participants, who were randomly assigned to one of the NMES groups, were blinded for the type of stimulation frequency.

### Quadriceps muscle function

Quadriceps muscle function (i.e., peak muscle strength and muscle endurance), using a Biodex (Biodex System 4 Pro, Biodex Medical Systems, Inc., New York, USA). Quadriceps peak muscle strength (Newton-meter, Nm) and quadriceps muscle endurance (Joules, J) were measured isokinetically. The participants performed thirty volitional maximal contractions at an angular velocity of 90° per second. *See* Additional file 1 for all details concerning the interventions.

### Functional exercise performance

Functional exercise performance was measured with the 6-min walk test, including a practice walk at initial assessment [23]. The best 6-min walk distance (6MWD) was used for further analyses. The constant work-rate cycling endurance test (CWRT, expressed in seconds) was performed at 75% of the pre-determined peak cycling rate, which has a high reliability in individuals with COPD [24]. Symptom scores for exercise-induced dyspnea and fatigue were assessed before and after these exercise tests.

### Metabolic load

Continuous on-line calculations of breath-by-breath oxygen uptake (VO_2_) and minute ventilation (V_E_), heart rate and oxygen saturation were obtained using the Oxycon mobile, a portable metabolic system (Carefusion the Netherlands, Houten, the Netherlands). The metabolic load was measured during sessions of HF-NMES, LF-NMES, or strength training in the first week, the fourth week, and in the last week of the trial. This methodology has been used before in individuals with COPD [14,15]. *See* Additional file 1 for all details concerning the measurement of the metabolic load.

### Statistical analysis

Analyses were performed using SPSS for Windows, Version 17.0.1 (SPSS, Inc., Chicago, Il, USA). Descriptive statistics were presented as median and interquartile range unless otherwise stated. Differences within groups were analyzed using Wilcoxon signed rank test. Groups were compared using Mann–Whitney U test or Kruskal-Wallis one-way analysis of variance. All tests were two-sided using a significance level of 5%.

## Results

### Baseline characteristics

**Figure 1:**
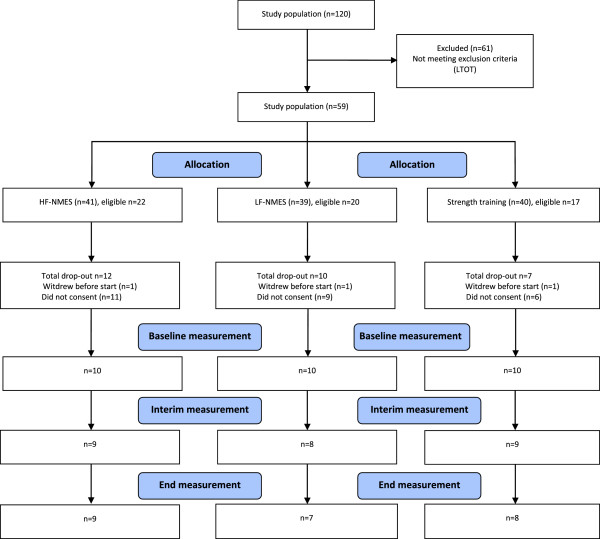
Flow diagram of measurements metabolic load.

Of the 120 individuals who participated in the ***DICES*** trial, 61 individuals (51%) were ineligible for the additional metabolic data collection due to the use of long-term oxygen therapy. Moreover, 26 individuals (22%) did not consent to this extra set of tests and 3 individuals (2%) who gave informed consent withdrew before start. At baseline, from 30 individuals (25%) intervention-related peak VO_2_, VE, heart rate and oxygen saturation were obtained. From 24 individuals the metabolic load was measured during a session of HF-NMES (n = 9), LF-NMES (n = 7), or strength training (n = 8) in the first week, the fourth week, and in the last week of the trial (Figure 1).

The 24 individuals of this pre-specified sub-analysis had severe to very severe COPD, a poor diffusing capacity for carbon monoxide and a poor functional and peak exercise performance (Table 1). Besides age and the relative peak ventilation during the cardiopulmonary exercise test, baseline characteristics did not significantly differ between intervention groups (Table 1). Mean FEV_1_ (34 ± 2% versus 31 ± 1% pred), PaCO2 (5.2 ± 0.1 kPa versus 5.8 ± 0.1 kPa), fat-free mass index (17.2 ± 0.4 kg/m^2^ versus 16.3 ± 0.2 kg/m^2^) and total work (1389 ± 95 J versus 1122 ± 49 J) were significantly higher in the metabolic load group compared to the remaining group, other baseline characteristics (age, DLco, residual volume, PaO_2_, SO_2_, peak and functional exercise performance, body mass index, peak torque, HADS anxiety and depression and health status) were not significantly different between both groups (Additional file 1: Table S1).

**Table 1:** General characteristics

	**Total group**	**HF-NMES**	**LF-NMES**	**Strength training**	**P-value**
	**n = 24**	**n = 9**	**n = 7**	**n = 8**	
Sex (M/F)	14/10	6/3	4/173	4/4	0.791
Age (years)	66 (61–68)	64 (59–67)	62 (59–67)	71 (66–77)	0.012
Pulmonary function					
FEV_1_ (liters )	0.98 (0.78-1.05)	1.01 (0.84-1.19)	0.74 (0.60-0.84)	1.00 (0.96-1.10)	0.010
FEV_1_ (% predicted)	35 (29–51)	34 (23–51)	30 (20–38)	46 (43–60)	0.064
FEV_1_/VC max (%)	32 (24–43)	31 (24–41)	25 (24–29)	42 (33–52)	0.057
DL_CO_ (%)	42 (36–55)	41 (31–55)	42 (34–61)	46 (40–56)	0.802
RV (%)	177 (136–235)	190 (137–243)	208 (172–260)	146 (128–178)	0.081
Arterial blood gases					
PaO_2_ (kPa)	9.8 (8.8-10.8)	9.0 (8.5-10.9)	9.6 (8.5-9.9)	10.3 (9.4-11.2)	0.284
PaCO_2_ (kPa)	4.9 (4.7-5.5)	5.4 (4.8-5.7)	5.0 (4.7-5.6)	4.8 (4.6-5.1)	0.159
SaO_2_ (%)	96 (95–97)	95 (95–97)	95 (94–97)	97 (96–98)	0.166
GOLD classification (I/II/III/IV)	0/7/10/7	0/3/3/3	0/0/4/3	0/4/3/1	0.025
GOLD classification (new) (A/B/C/D)	0/3/0/21	0/2/0/7	0/0/0/7	0/1/0/7	0.427
BMI (kg/m^2^)	25.4 (22.2-29.8)	25.4 (21.9-26.9)	24.6 (23.5-30.3)	26.9 (21.5-31.1)	0.834
FFMI (kg/m^2^)	17.0 (15.8-18.5)	16.9 (15.7-17.5)	16.5 (15.7-18.8)	17.4 (15.8-19.1)	0.680
Cardiopulmonary exercise test					
Peak load (watts)	47 (35–57)	51 (35–57)	46 (41–62)	44 (30–58)	0.867
Peak load (% predicted)	33 (23–47)	26 (23–39)	38 (22–81)	39 (23–63)	0.481
Peak VO_2_ ( ml/min)	841 (710–987)	751 (697–919)	817 (734–1087)	904 (699–1076)	0.473
Peak VO_2_ (% predicted)	48 (33–73)	31 (27–64)	53 (40–90)	52 (40–115)	0.142
Peak VE (liters)	33 (29–39)	38 (28–41)	32 (25–35)	35 (29–44)	0.553
Peak VE (% MVV)	89 (78–107)	84 (72–98)	103 (95–119)	81 (68–101)	0.040
Peak HR (bpm)	104 (98–124)	103 (98–122)	126 (104–131)	102 (90–109)	0.080
Peak HR (% predicted)	71 (64–75)	66 (64–73)	75 (68–83)	70 (63–72)	0.186
Dyspnea, end (points)	7 (5–8)	7 (5–7.5)	7 (7–9)	8 (5–9.5)	0.526
Fatigue, end (points)	5 (3–7)	5 (3.5-7.5)	5 (3–7)	4 (3–7)	0.861
Saturation, end (%)	91 (89–95)	93 (91–98)	90 (89–91)	94 (89–96)	0.132

Values expressed as median (interquartile range) or numbers. Data are compared between HF-NMES, LF-NMES and strength training.

*Abbreviations:* HF-NMES = High-frequency transcutaneous neuromuscular electrical stimulation; LF-NMES = Low-frequency transcutaneous neuromuscular electrical stimulation; M = males; F = females; FEV_1_ = forced expiratory volume in one second; VC max = maximum vital capacity; DL_CO_ = diffusion capacity of the lung for carbon monoxide; RV = residual volume; PaO2 = resting arterial oxygen tension; PaCO2 = resting arterial carbon dioxide tension; SaO2 = resting arterial oxygen tension; kPa = kilopascal; LTOT = long-term oxygen therapy; GOLD = Global Initiative on Obstructive Lung Diseases; BMI = body mass index; FFMI = fat free mass index; kg/m^2^ = kilogram per square meter; VO_2_ = oxygen uptake; tSaO_2_ = transcutaneous oxygen saturation; ml/min = milliliters per minute; % MVV = percentage maximal voluntary ventilation; bpm = beats per minute.

### Efficacy of interventions

#### Quadriceps muscle strength

**Figure 2:**
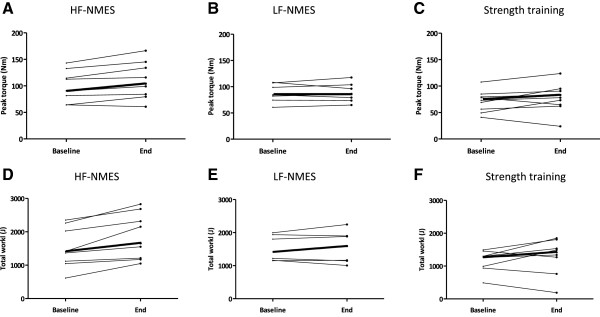
**Individual changes in quadriceps peak torque and total work from baseline to end.** Abbreviations: HF-NMES=high-frequency transcutaneous neuromuscular electrical stimulation; LF-NMES=low-frequency transcutaneous neuromuscular electrical stimulation (quadriceps peak torque: **A, B** and **C**; quadriceps muscle endurance: **D, E** and **F**). The bold line represents the median values.

Isokinetic quadriceps peak torque increased significantly following HF-NMES (12.6 Nm (3.0-17.5 Nm); p = 0.021), but not following LF-NMES (4.2 Nm (−5.2-5 Nm); p = 0.866) or strength training (5.7 Nm (−9-22 Nm); p = 0.263) (Figure 2). There were no significant between-group differences in changes.

#### Quadriceps muscle endurance

Isokinetic total work increased significantly following HF-NMES (292 J (156–501 J); p < 0.01), but not following LF-NMES (12 J (−73-178 J); p = 0.499) or strength training (157 J (−185-456 J); p = 0.263) (Figure 2). The improvement following HF-NMES was significantly higher compared to LF-NMES (p = 0.005) (Figure 2).

#### Six-minute walk distance

6MWD improved significantly following HF-NMES (75 m (29–138 m); p = 0.008) or strength training (57 m (32–85 m); p = 0.034), but not following LF-NMES (27 m (−34-65 m); p = 0.310). There were no significant differences in changes between groups.

#### Constant work-rate test

Endurance time during the constant work-rate cycling test improved significantly following HF-NMES (92 s (49–275 s); p = 0.066), LF-NMES (83 s (−7-223 s); p = 0.091) or strength training (37 s (1–98 s); p = 0.043). There were no significant differences in changes between groups.

### Course of HF-NMES, LF-NMES or strength training

**Figure 3:**
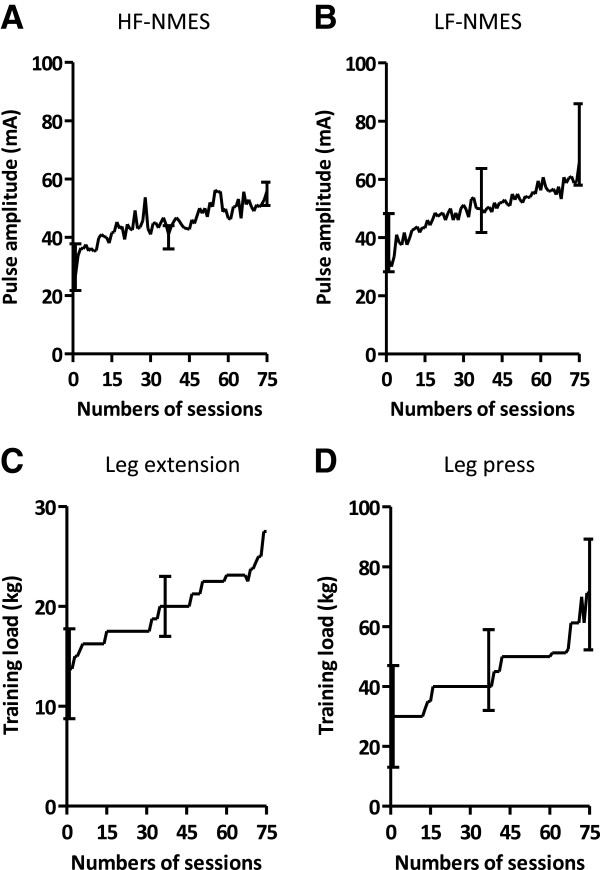
**Course in pulse amplitude and training load during the rehabilitation program.** Course in pulse amplitude is shown during HF-NMES **(A)** and LF-NMES **(B)**. Course in training load is shown during leg extension exercise **(C)** and leg press **(D)**. Data are shown as median and interquartile range. Abbreviations: HF-NMES=high-frequency transcutaneous neuromuscular electrical stimulation; LF-NMES=low-frequency transcutaneous neuromuscular electrical stimulation.

In the HF-NMES group, pulse amplitude ranged from 12 to 40 mA in week one to 34 to 71 mA in week eight (p = 0.008); and in the LF-NMES group from 25 to 50 mA to 35 to 98 mA (p = 0.063, Figure 3). Median end dyspnea scores, end fatigue scores and end muscle pain scores ranged from 1 to 3 points, from 0.5 to 2 points and from 0 to 6 points in the HF-NMES group and from 2 to 3 points, from 2 to 5 points and from 0 to 9 points in the LF-NMES group respectively. The load during leg extension strengthening exercise ranged from 2.5 to 20 kg in week one to 12.5 to 27.5 kg in week eight; and from 5 to 70 kg to 35 to 90 kg for the leg press strengthening exercise (both p < 0.02, Figure 3). In addition, median end dyspnea scores, end fatigue scores and end muscle pain scores ranged from 2 to 5 points, from 1.5 to 4 points and from 0 to 28 points in the strength training group.

### Metabolic load during HF-NMES, LF-NMES or strength training

**Figure 4:**
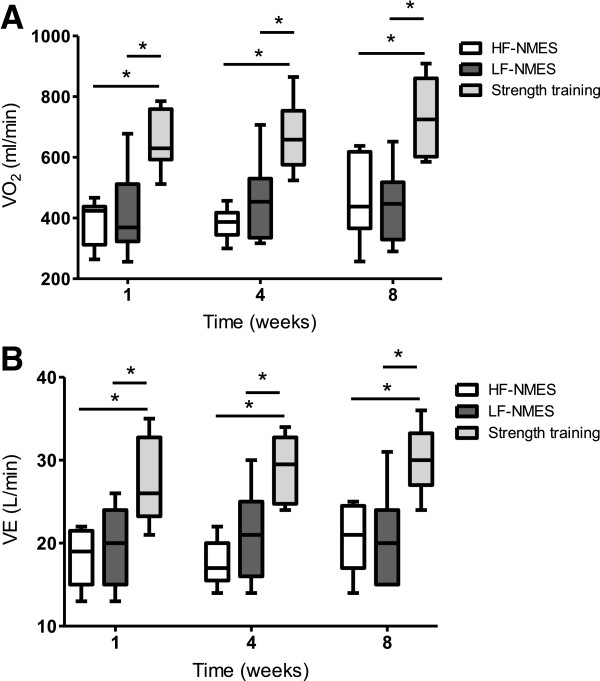
**Course in VO**_**2 **_**and ventilation during HF-NMES, LF-NMES and strength training.** Course in VO_2_ is shown in **A** and course in ventilation (VE) is shown in **B**. Data are shown as median and interquartile range. Abbreviations: HF-NMES=high-frequency transcutaneous neuromuscular electrical stimulation; LF-NMES=low-frequency transcutaneous neuromuscular electrical stimulation; VO_2_=oxygen uptake. *p<0.05.

Intervention-related peak VO_2_ did not change over time all three interventions (Figure 4A, Table 2). Intervention-related peak V_E_ did not change over time in the strength training or LF-NMES group (p > 0.171). In the HF-NMES group, the intervention-related V_E_ increased slightly, but significantly over time (p = 0.012, Figure 4B). At all measurement points, intervention-related peak VO_2_ and V_E_ were significantly higher during strength training sessions compared to the HF-NMES or LF-NMES sessions (all p < 0.05). There were no differences between HF-NMES and LF-NMES (p > 0.28). Oxygen saturation (lowest values) was significant lower at the end of the fist measurements in the HF-NMES group and the strength training group compared with baseline (both p < 0.03), during the other measurements no significant changes in oxygen saturation were observed (all p > 0.05) (Table 3). There were no significant between-group differences in changes in oxygen saturation (all p > 0.05). Heart rate was significantly higher at the end compared to baseline in all groups in all measurements (p < 0.05) (Table 3). During all measurements, changes in heart rate were significantly higher after strength training compared to HF-NMES or LF-NMES (p < 0.04).

**Table 2:** Oxygen uptake and ventilation during HF-NMES, LF-NMES or strength training

		**HF-NMES**	**LF-NMES**	**Strength training**
		**n = 9**	**n = 8**	**n = 7**
VO_2_	ml/min	424 (312–438)	369 (323–512)	630 (593–759)
VE	L/min	19 (15–22)	20 (15–24)	26 (23–33)
VO_2_	ml/min	387 (345–418)	454 (335–530)	659 (576–754)
VE	L/min	17 (16–20)	21 (16–25)	30 (25–33)
VO_2_	ml/min	438 (366–619)	447 (329–518)	725 (603–861)
VE	L/min	21 (17–25)	20 (15–24)	30 (27–33)

Values expressed as median (interquartile range).

*Abbreviations:* HF-NMES = high-frequency transcutaneous neuromuscular electrical stimulation; LF-NMES = low-frequency transcutaneous neuromuscular electrical stimulation; VO_2_ = oxygen uptake; VE = ventilation; ml/min = milliliters per minute; L/min = liters per minute.

**Table 3:** Oxygen saturation and peak heart rate during HF-NMES, LF-NMES or strength training

		**HF-NMES**			**LF-NMES**			**Strength training**		
		**n = 9**			**n = 8**			**n = 7**		
		**Pre**	**Post**	**P-value**	**Pre**	**Post**	**P-value**	**Pre**	**Post**	**P-value**
First measurement										
SpO_2_	%	96 (92–97)	93 (91–95)	0.020	89 (86–96)	88 (84–95)	0.059	95 (89–95)	91 (84–93)	0.027
Heart rate	bpm	83 (77–87)	92 (82–97)	0.007	95 (80–106)	100 (85–112)	0.042	66 (60–91)	86 (77–102)	0.018
Second measurement										
SpO_2_	%	93 (88–97)	91 (88–95)	0.056	94 (92–98)	92 (85–96)	0.066	92 (90–94)	88 (88–91)	0.149
Heart rate	bpm	83 (78–89)	91 (85–100)	0.012	87 (85–97)	100 (93–106)	0.018	74 (58–89)	93 (75–109)	0.012
Third measurement										
SpO_2_	%	93 (89–98)	93 (89–97)	0.666	87 (85–96)	92 (84–94)	0.498	95 (91–96)	91 (88–94)	0.058
Heart rate	bpm	82 (73–90)	91 (88–101)	0.008	84 (77–98)	94 (82–106)	0.043	76 (62–93)	93 (79–109)	0.018

Values expressed as median (interquartile range).

*Abbreviations:* HF-NMES = high-frequency transcutaneous neuromuscular electrical stimulation; LF-NMES = low-frequency transcutaneous neuromuscular electrical stimulation; SpO_2_ = oxygen saturation; bpm = beats per minute.

## Discussion

This is the first study to investigate the metabolic load of various local muscle training modalities in severely dyspneic COPD patients with quadriceps muscle weakness during the course of an inpatient pulmonary rehabilitation program. It showed that the metabolic load measured during successive sessions of strength training, HF-NMES, or LF-NMES remained generally stable, while the NMES pulse amplitude or the strength training load increased significantly during the eight-week intervention period.

The median oxygen uptake during the baseline session of HF-NMES, LF-NMES or strength training ranged between 30 and 99% of the peak aerobic capacity measured during the cardiopulmonary exercise test, and was comparable with previous studies [7,14,15]. The median oxygen uptake was 99% of the peak aerobic capacity in one subject who was characterized with very severe COPD (FEV_1_: 17% pred) and a very decreased exercise capacity (peak load: 20% pred; VO_2_ peak: 688 ml/min (26% pred)). At all measurement points, intervention-related peak VO_2_ and V_E_ were significantly lower during NMES (LF and HF) compared with strength training. This is in line with previous studies measuring the metabolic load in patients with COPD during NMES or strength training [14,15]. However, these results are in contrast with the findings of a study in healthy male recreational athletes comparing one session of voluntary contractions with one session of HF-NMES (75 Hz) [25]. Theurel and colleagues found that the average oxygen consumption and ventilation were significantly higher during HF-NMES compared with voluntary exercise [25]. Besides the study design and subjects, an important difference with the present study is the training load [25]. Theurel and colleagues used an average training load in strength training of 46% of the maximal voluntary contraction instead of 60-70% of the one-repetition maximum which is used in our study and what is recommended by the American College of Sports Medicine [8].

The present study shows no changes over time in the metabolic load which is not in line with the study of Probst and colleagues [7]. Probst and colleagues showed a significant increase in oxygen uptake and ventilation during a 12 wk program of leg press exercises [7]. This could be attributable to a greater increase in the training load. However, the change in training load of leg press exercises was comparable to the present study.

The median increase in pulse amplitude during the study was 24 mA in HF-NMES and 37 mA in LF-NMES. The course in pulse amplitude is comparable with previous studies in severely disabled patients with COPD which respond to NMES [16,26]. The low metabolic load accompanied with acceptable low dyspnea and fatigue scores probably explains the applicability of these interventions in severely disabled and dyspneic patients, even during acute COPD exacerbations [26-28]. This is also probably the reason that NMES can easily be applied in bed-bound individuals with chronic hypercapnic respiratory failure due to COPD who are receiving mechanical ventilation [29] or critically ill patients in the intensive care unit [30].

Because of the constant metabolic load in combination with stable symptom scores over time, it seems reasonable to hypothesize that the improvements in muscle function are at least partially due to intramuscular changes. Previously, it has been shown that type I and IIa fibers increased following LF-NMES [31,32] or HF-NMES [33,34]. Strength training generally results in increased levels of glycolytic enzymes [35] and an increase in percentage and size of type II fibers [36-39].

Obviously, this study has some limitations. First, the inclusion criteria of the *DICES* trial limits the external validity of the present findings. Only COPD patients with an mMRC score of 3 and 4 in combination with muscle weakness were included. Secondly, the small sample size and selected patient characteristics for participation in the measurements with the Oxycon mobile may be an important reason for detecting no significant improvements in peak muscle strength in the strength training group and quadriceps muscle endurance in the LF-NMES group and the strength training group. Only patients without long-term oxygen therapy (LTOT) were eligible to participate due to the methodology used [7], although LTOT patients are also likely to have benefit from these interventions. However, the equipment (Oxycon mobile, a portable metabolic system) is not able to measure oxygen uptake (VO_2_) while breathing inspiratory O_2_ fractions [7]. In the DICES trial 51% of the patients used LTOT [40]. It is unclear if the metabolic load might be different in LTOT patients. Moreover, the small sample size and the exclusion of LTOT patients may limit the external validity and broad applicability of these findings.

## Conclusion

To conclude, the metabolic load and symptom scores for dyspnea, fatigue and muscle pain remain acceptable low over time with increasing training loads during HF-NMES, LF-NMES or strength training. For this reason, these interventions are recommended in severely dyspneic patients with COPD for improving their muscle function and exercise performance.

## Competing interests

The authors declare that they have no competing interests.

## Authors’ contributions

Study concept and design: MJHS, EFMW and MAS; acquisition of data: MJHS, JMLD, AWV; analysis and interpretation of data: MJHS, FMEF and MAS; drafting the article: MJHS, FMEF and MAS; revising it critically for important intellectual content: all authors; final approval of the version to be published: all authors. MJHS had full access to all study data and takes responsibility for the integrity of the data and the accuracy of the data analysis.

## Pre-publication history

The pre-publication history for this paper can be accessed here:

http://www.biomedcentral.com/1471-2466/14/146/prepub

## Supplementary Material

Additional file 1 Online Supplement. Click here for file

## References

[B1] Seymour JM Spruit MA Hopkinson NS Natanek SA Man WD Jackson A Gosker HR Schols AM Moxham J Polkey MI Wouters EF The prevalence of quadriceps weakness in COPD and the relationship with disease severity Eur Respir J 2010 36 1 81 88 10.1183/09031936.00104909 19897554 PMC3039205

[B2] Spruit MA Pennings HJ Janssen PP Does JD Scroyen S Akkermans MA Mostert R Wouters EF Extra-pulmonary features in COPD patients entering rehabilitation after stratification for MRC dyspnea grade Respir Med 2007 101 12 2454 2463 10.1016/j.rmed.2007.07.003 17765532

[B3] Spruit MA Watkins ML Edwards LD Vestbo J Calverley PM Pinto-Plata V Celli BR Tal-Singer R Wouters EF Determinants of poor 6-min walking distance in patients with COPD: the ECLIPSE cohort Respir Med 2010 104 6 849 857 10.1016/j.rmed.2009.12.007 20471236

[B4] Waschki B Spruit MA Watz H Albert PS Shrikrishna D Groenen M Smith C Man WD Tal-Singer R Edwards LD Calverley PM Magnussen H Polkey MI Physical activity monitoring in COPD: compliance and associations with clinical characteristics in a multicenter study Respir Med 2012 106 4 522 530 10.1016/j.rmed.2011.10.022 22118987

[B5] Spruit MA Singh SJ Garvey C Zuwallack R Nici L Rochester C Hill K Holland AE Lareau SC Man WD Pitta F Sewell L Raskin J Bourbeau J Crouch R Franssen FM Casaburi R Vercoulen JH Vogiatzis I Gosselink R Clini EM Effing TW Maltais F van der Palen J Troosters T Janssen DJ Collins E Garcia-Aymerich J Brooks D Fahy BF An official american thoracic society/european respiratory society statement: key concepts and advances in pulmonary rehabilitation Am J Respir Crit Care Med 2013 188 8 e13 e64 10.1164/rccm.201309-1634ST 24127811

[B6] Hogg L Garrod R Thornton H McDonnell L Bellas H White P Effectiveness, attendance, and completion of an integrated, system-wide pulmonary rehabilitation service for COPD: prospective observational study Copd 2012 9 5 546 554 10.3109/15412555.2012.707258 23030586

[B7] Probst VS Troosters T Pitta F Decramer M Gosselink R Cardiopulmonary stress during exercise training in patients with COPD Eur Respir J 2006 27 6 1110 1118 10.1183/09031936.06.00110605 16540501

[B8] American College of Sports Medicine position stand Progression models in resistance training for healthy adults Med Sci Sports Exerc 2009 41 3 687 708 10.1249/MSS.0b013e3181915670 19204579

[B9] Maffiuletti NA Physiological and methodological considerations for the use of neuromuscular electrical stimulation Eur J Appl Physiol 2010 110 2 223 234 10.1007/s00421-010-1502-y 20473619

[B10] Vanderthommen M Duchateau J Electrical stimulation as a modality to improve performance of the neuromuscular system Exerc Sport Sci Rev 2007 35 4 180 185 10.1097/jes.0b013e318156e785 17921786

[B11] Spruit MA Wouters EF New modalities of pulmonary rehabilitation in patients with chronic obstructive pulmonary disease Sports Med 2007 37 6 501 518 10.2165/00007256-200737060-00004 17503876

[B12] Sillen MJ Speksnijder CM Eterman RM Janssen PP Wagers SS Wouters EF Uszko-Lencer NH Spruit MA Effects of neuromuscular electrical stimulation of muscles of ambulation in patients with chronic heart failure or COPD: a systematic review of the English-language literature Chest 2009 136 1 44 61 10.1378/chest.08-2481 19363213

[B13] Sillen MJ Franssen FM Delbressine JM Vaes AW Wouters EF Spruit MA Efficacy of lower-limb muscle training modalities in severely dyspnoeic individuals with COPD and quadriceps muscle weakness: results from the DICES trial Thorax 2014 69 6 525 531 10.1136/thoraxjnl-2013-204388 24399630

[B14] Sillen MJ Janssen PP Akkermans MA Wouters EF Spruit MA The metabolic response during resistance training and neuromuscular electrical stimulation (NMES) in patients with COPD, a pilot study Respir Med 2008 102 5 786 789 10.1016/j.rmed.2008.01.013 18294832

[B15] Sillen MJ Wouters EF Franssen FM Meijer K Stakenborg KH Spruit MA Oxygen uptake, ventilation, and symptoms during low-frequency versus high-frequency NMES in COPD: a pilot study Lung 2011 189 1 21 26 10.1007/s00408-010-9265-0 21080183

[B16] Vivodtzev I Debigare R Gagnon P Mainguy V Saey D Dube A Pare ME Belanger M Maltais F Functional and muscular effects of neuromuscular electrical stimulation in patients with severe COPD: a randomized clinical trial Chest 2012 141 3 716 725 10.1378/chest.11-0839 22116795

[B17] Borges O Isometric and isokinetic knee extension and flexion torque in men and women aged 20–70 Scand J Rehabil Med 1989 21 1 45 53 2711137

[B18] Spruit MA Gosselink R Troosters T De Paepe K Decramer M Resistance versus endurance training in patients with COPD and peripheral muscle weakness Eur Respir J 2002 19 6 1072 1078 10.1183/09031936.02.00287102 12108859

[B19] Revill SI Robinson JO Rosen M Hogg MI The reliability of a linear analogue for evaluating pain Anaesthesia 1976 31 9 1191 1198 10.1111/j.1365-2044.1976.tb11971.x 1015603

[B20] Wegmann H The “new” Declaration of Helsinki J Int Biotechnol Law 2009 6 4 173 176

[B21] Sillen MJ Franssen FM Delbressine JM Uszko-Lencer NH Vanfleteren LE Rutten EP Wouters EF Spruit MA Heterogeneity in clinical characteristics and co-morbidities in dyspneic individuals with COPD GOLD D: findings of the DICES trial Respir Med 2013 107 8 1186 1194 10.1016/j.rmed.2013.04.020 23706780

[B22] Sillen MJ Franssen FM Delbressine JM Vaes AW Wouters EF Spruit MA Efficacy of lower-limb muscle training modalities in severely dyspnoeic individuals with chronic obstructive pulmonary disease and quadriceps muscle weakness: results from the DICES trial Thorax 2013 in press 10.1136/thoraxjnl-2013-204388 24399630

[B23] Hernandes NA Wouters EF Meijer K Annegarn J Pitta F Spruit MA Reproducibility of 6-minute walking test in patients with COPD Eur Respir J 2011 38 2 261 267 10.1183/09031936.00142010 21177838

[B24] Hul van ’t A Gosselink R Kwakkel G Constant-load cycle endurance performance: test-retest reliability and validity in patients with COPD J Cardiopulm Rehabil 2003 23 2 143 150 10.1097/00008483-200303000-00012 12668937

[B25] Theurel J Lepers R Pardon L Maffiuletti NA Differences in cardiorespiratory and neuromuscular responses between voluntary and stimulated contractions of the quadriceps femoris muscle Respir Physiol Neurobiol 2007 157 2–3 341 347 10.1016/j.resp.2006.12.002 17210271

[B26] Giavedoni S Deans A McCaughey P Drost E MacNee W Rabinovich RA Neuromuscular electrical stimulation prevents muscle function deterioration in exacerbated COPD: a pilot study Respir Med 2012 106 10 1429 1434 10.1016/j.rmed.2012.05.005 22726566

[B27] Abdellaoui A Prefaut C Gouzi F Couillard A Coisy-Quivy M Hugon G Molinari N Lafontaine T Jonquet O Laoudj-Chenivesse D Hayot M Skeletal muscle effects of electrostimulation after COPD exacerbation: a pilot study Eur Respir J 2011 38 4 781 788 10.1183/09031936.00167110 21349913

[B28] Troosters T Probst VS Crul T Pitta F Gayan-Ramirez G Decramer M Gosselink R Resistance training prevents deterioration in quadriceps muscle function during acute exacerbations of chronic obstructive pulmonary disease Am J Respir Crit Care Med 2010 181 10 1072 1077 10.1164/rccm.200908-1203OC 20133927

[B29] Zanotti E Felicetti G Maini M Fracchia C Peripheral muscle strength training in bed-bound patients with COPD receiving mechanical ventilation: effect of electrical stimulation Chest 2003 124 1 292 296 10.1378/chest.124.1.292 12853536

[B30] Maffiuletti NA Roig M Karatzanos E Nanas S Neuromuscular electrical stimulation for preventing skeletal-muscle weakness and wasting in critically ill patients: a systematic review BMC Med 2013 11 137 10.1186/1741-7015-11-137 PMC3668245 23701811

[B31] Nuhr M Crevenna R Gohlsch B Bittner C Pleiner J Wiesinger G Fialka-Moser V Quittan M Pette D Functional and biochemical properties of chronically stimulated human skeletal muscle Eur J Appl Physiol 2003 89 2 202 208 10.1007/s00421-003-0792-8 12665986

[B32] Theriault R Boulay MR Theriault G Simoneau JA Electrical stimulation-induced changes in performance and fiber type proportion of human knee extensor muscles Eur J Appl Physiol Occup Physiol 1996 74 4 311 317 10.1007/BF02226926 8911822

[B33] Gondin J Brocca L Bellinzona E D’Antona G Maffiuletti NA Miotti D Pellegrino MA Bottinelli R Neuromuscular electrical stimulation training induces atypical adaptations of the human skeletal muscle phenotype: a functional and proteomic analysis J Appl Physiol 2011 110 2 433 450 10.1152/japplphysiol.00914.2010 21127206

[B34] Perez M Lucia A Rivero JL Serrano AL Calbet JA Delgado MA Chicharro JL Effects of transcutaneous short-term electrical stimulation on M. vastus lateralis characteristics of healthy young men Pflugers Arch 2002 443 5–6 866 874 10.1007/s00424-001-0769-6 11889587

[B35] Tesch PA Thorsson A Essen-Gustavsson B Enzyme activities of FT and ST muscle fibers in heavy-resistance trained athletes J Appl Physiol 1989 67 1 83 87 10.1152/jappl.1989.67.1.83 2547751

[B36] Kryger AI Andersen JL Resistance training in the oldest old: consequences for muscle strength, fiber types, fiber size, and MHC isoforms Scand J Med Sci Sports 2007 17 4 422 430 10.1111/j.1600-0838.2006.00575.x 17490465

[B37] Andersen JL Aagaard P Myosin heavy chain IIX overshoot in human skeletal muscle Muscle Nerve 2000 23 7 1095 1104 10.1002/1097-4598(200007)23:7<1095::AID-MUS13>3.0.CO;2-O 10883005

[B38] Farup J Kjolhede T Sorensen H Dalgas U Moller AB Vestergaard PF Ringgaard S Bojsen-Moller J Vissing K Muscle morphological and strength adaptations to endurance vs. resistance training J Strength Cond Res 2012 26 2 398 407 10.1519/JSC.0b013e318225a26f 22266546

[B39] Verdijk LB Gleeson BG Jonkers RA Meijer K Savelberg HH Dendale P van Loon LJ Skeletal muscle hypertrophy following resistance training is accompanied by a fiber type-specific increase in satellite cell content in elderly men J Gerontol A Biol Sci Med Sci 2009 64 3 332 339 10.1093/gerona/gln050 PMC2655000 19196907

[B40] Sillen MJ Franssen FM Delbressine JM Vaes AW Wouters EF Spruit MA Efficacy of lower-limb muscle training modalities in severely dyspnoeic individuals with COPD and quadriceps muscle weakness: response from the authors Thorax 2014 doi: 10.1136/thoraxjnl-2014-205781 10.1136/thoraxjnl-2014-205781 24928811

