# Supplementary material for: Metabolic load during strength training or NMES in individuals with COPD: results from the DICES trial
Source: BMC Pulm Med. 2014 Sep 2;14:146. doi: 10.1186/1471-2466-14-146 (PMC4236758; doi:10.1186/1471-2466-14-146)
Supplement: Additional file 1 — Online Supplement. [file 1471-2466-14-146-S1.doc]

**Additional file 1: Online Supplement**

**Metabolic load during strength training or NMES in individuals with COPD: results from the DICES trial**

**Interventions**

The ***DICES*** trial was part of a regular eight-week inpatient pulmonary rehabilitation program, including also non-exercising components like occupational therapy, exacerbation management strategies, relaxation therapy, educational sessions, and psychosocial counselling. The interdisciplinary treatment was comparable amongst groups. None of the participants underwent treadmill walking or stationary ergometry cycling.

Lower-limb muscle training existed of one of the following interventions: HF-NMES; LF-NMES; or strength training. The interventions took place in group sessions, twice per day, 5 times per week for 8 weeks. All sessions were supervised by a physiotherapist. Symptom scores for dyspnoea, fatigue, and muscle pain were assessed before and directly after each session.

*NMES protocols*

NMES involves the application of an electrical current through electrodes placed on the skin over the targeted muscles, thereby depolarizing motor neurons and, in turn, inducing skeletal muscle contractions. Quadriceps and calf muscles of both legs were stimulated electrically with a portable battery-operated electrical stimulator (Tensmed S84, Enraf-Nonius, Rotterdam, the Netherlands) (figure E1). The output characteristics of the device have been checked on an oscilloscope. A total of eight carbon-rubber electrodes in moistened sponges were placed on the target muscles (four electrodes on each leg): two pairs of 8 x 12 cm on the quadriceps muscles and two pairs of 4 x 6 cm on the calf muscles. The electrodes on the quadriceps femoris muscles were placed transversally 5-10 cm distal to the inguinal fold and 4-8 cm proximal to the patella. The electrodes on the calf muscles were placed longitudinally on the belly of the gastrocnemii muscles. Both NMES protocols used a symmetrical biphasic square pulse with pulse duration of 400 μs. The contraction time was 6 seconds with 8 seconds relaxation excluding 1 second ramp-up and 1 second ramp-down. Thus, the total cycle length was 16 seconds. After a continuous warm-up of 3 minutes at 5 Hz, intensity was adjusted to individual toleration during each session lasting 18 minutes. The frequencies used were 75 Hz (HF-NMES) or 15 Hz (LF-NMES).

*Strength training*

Strength training involves exercises that cause muscles to work or hold against an externally applied force or weight. Strength training consisted of bilateral leg extension and bilateral leg press exercises (Technogym SpA, Gambettola, Italy). The 1RM was determined during the initial assessment to target the training load. Both exercises started at 70% of 1-repetition maximum (1RM), 4 sets of 8 repetitions per exercise with at least 2 minutes of recovery between each set. The training load was set to increase with 5% every two weeks.

**Outcomes**

Quadriceps muscle function

The primary outcome parameter was the change in isokinetic quadriceps muscle function (i.e., peak muscle strength and muscle endurance), using a Biodex (Biodex System 4 Pro, Biodex Medical Systems, Inc., New York, USA). The reliability of this method has been demonstrated previously in patients with COPD. To avoid learning effects, the measurement was performed twice at the initial assessment. Best values were used for further analyses. During quadriceps muscle function testing, participants were seated upright on the chair of the dynamometer with support of the back and an angle of 90^0^ of flexion in the hip joint. The participants were secured with straps. The lever arm was attached to the distal part of the tibia and its axis of rotation was aligned with the anatomical axis of the knee joint. Subjects were instructed to keep their hands on their thighs during testing and were asked to perform maximum strength. The participants performed thirty sequential volitional maximal contractions at an angular velocity of 90^0^ per second. They were strongly encouraged during this isokinetic test. Peak quadriceps muscle strength was defined as the highest peak torque (Newton-meter, Nm) and quadriceps muscle endurance as the total amount of delivered work (Joules, J) in this series of thirty contractions.

Metabolic load

During both NMES sessions continuous on-line calculations of breath-by-breath oxygen uptake (VO_2_) and minute ventilation (V_E_) were obtained using the Oxycon mobile, a portable metabolic system (Carefusion the Netherlands, Houten, the Netherlands). After calibration the face mask (Combitox, Dräger Safety, Lübeck, Germany) was carefully adjusted to the patient’s face and checked for air leaks. Data were processed in the PC-software (JLAB version 5.20b, Carefusion the Netherlands, Houten, the Netherlands) and were collected breath by breath.

|  | | Metabolic load group  n=24 | Remaining group  n=96 | P-value |
| --- | --- | --- | --- | --- |
| Gender | Male/Female | 14/10 | 48/48 | 0.467 |
| Age | years | 65.3 + 1.5 | 64.7 + 0.9 | 0.765 |
| FEV_1_ | liters | 0.95 + 0.05 | 0.82 + 0.04 | 0.104 |
| FEV_1_ | % predicted | 38 + 3 | 32 + 1 | 0.048 |
| FEV_1_/VC max | % | 34 + 2 | 31 + 1 | 0.288 |
| DL_CO_ | % | 46 + 3 | 40 + 2 | 0.102 |
| RV | % | 186 + 12 | 202 + 6 | 0.207 |
| PaO_2_ | kPa | 9.7 + 0.2 | 9.7 + 0.2 | 0.932 |
| PaCO_2_ | kPa | 5.2 + 0.1 | 5.8 + 0.1 | 0.001 |
| SaO_2_ | % | 95.9 + 0.4 | 95.51 + 0.3 | 0.181 |
| Peak load | watts | 46 + 3 | 44 + 1 | 0.511 |
| Peak load | % predicted | 42 + 6 | 40 + 2 | 0.714 |
| Bodyweight | kg | 74 + 3 | 68 + 2 | 0.105 |
| BMI | kg/m^2^ | 26.1 + 1.0 | 24.5 + 0.5 | 0.171 |
| FFMI | kg/m^2^ | 17.2 + 0.4 | 16.3 + 0.2 | 0.037 |
| Peak torque | Nm | 86.6 + 5.2 | 73.6 + 2.7 | 0.030 |
| Peak torque | % predicted | 59 + 3 | 53 + 2 | 0.102 |
| Total work | Joules | 1389 + 95 | 1122 + 49 | 0.015 |
| 1RM leg extension | kg | 35 + 3 | 30 + 1 | 0.043 |
| 1RM leg press | kg | 78 + 8 | 66 + 3 | 0.134 |

**Table S1. Characteristics of metabolic load group and remaining group**

Values expressed as mean + SEM.

Abbreviations: FEV_1_=forced expiratory volume in one second; VC max=maximum vital capacity; DL_CO_=diffusion capacity of the lung for carbon monoxide; RV=residual volume; PaO_2_=resting arterial oxygen tension; PaCO_2_=resting arterial carbon dioxide tension; SaO_2_=resting arterial oxygen tension; BMI=body mass index; FFMI=fat-free mass index; 1RM= one-repetition maximum; Nm=newtonmeter; kPa= kilopascal; ml/min=milliliter per minute; kg/m^2^=kilogram per squared meter.

References

1. Spruit MA, Singh SJ, Garvey C, Zuwallack R, Nici L, Rochester C, Hill K, Holland AE, Lareau SC, Man WD *et al*: **An official american thoracic society/european respiratory society statement: key concepts and advances in pulmonary rehabilitation**. *Am J Respir Crit Care Med* 2013, **188**(8):e13-64.

2. Revill SI, Robinson JO, Rosen M, Hogg MI: **The reliability of a linear analogue for evaluating pain**. *Anaesthesia* 1976, **31**(9):1191-1198.

3. Vivodtzev I, Lacasse Y, Maltais F: **Neuromuscular electrical stimulation of the lower limbs in patients with chronic obstructive pulmonary disease**. *J Cardiopulm Rehabil Prev* 2008, **28**(2):79-91.

4. Vanderthommen M, Duchateau J: **Electrical stimulation as a modality to improve performance of the neuromuscular system**. *Exerc Sport Sci Rev* 2007, **35**(4):180-185.

5. Sillen MJ, Wouters EF, Franssen FM, Meijer K, Stakenborg KH, Spruit MA: **Oxygen uptake, ventilation, and symptoms during low-frequency versus high-frequency NMES in COPD: a pilot study**. *Lung* 2011, **189**(1):21-26.

6. Feigenbaum MS, Pollock ML: **Strength training: rationale for current guidelines for adult fitness programs**. *The Physician and sportsmedicine* 1997, **25**(2):44-63.

7. Ratamess NA, Alvar BA, Evetoch TK, Housh TJ, Kibler WB, Kraemer WJ, Triplett NT: **Progression Models in Resistance Training for Healthy Adults**. *Medicine & Science in Sports & Exercise* 2009:687-708.

8. Spruit MA, Gosselink R, Troosters T, De Paepe K, Decramer M: **Resistance versus endurance training in patients with COPD and peripheral muscle weakness**. *Eur Respir J* 2002, **19**(6):1072-1078.

9. Mathur S, Makrides L, Hernandez P: **Test-retest reliability of isometric and isokinetic torque in patients with chronic obstructive pulmonary disease.** *Physiother Can* 2004, **56**:94–101.

10. Franssen FM, Broekhuizen R, Janssen PP, Wouters EF, Schols AM: **Limb muscle dysfunction in COPD: effects of muscle wasting and exercise training**. *Med Sci Sports Exerc* 2005, **37**(1):2-9.
